# Growth Deceleration in Infants With Gastrointestinal Manifestations of Cow's Milk Allergy: Case Series From Specialised Centre

**DOI:** 10.1111/apa.70537

**Published:** 2026-04-10

**Authors:** Priscila Prazeres de Assis, Margarida Maria de Castro Antunes, Alcides da Silva Diniz, Adrielle Cavalcanti de Pontes Araújo, Camila de Souza Rêgo, Poliana Coelho Cabral

**Affiliations:** ^1^ Graduate Program in Nutrition Federal University of Pernambuco Recife Brazil; ^2^ Graduate Program in Child and Adolescent Healthcare Federal University of Pernambuco Recife Brazil; ^3^ Independent Researcher Recife Brazil

**Keywords:** cow's Milk allergy, elimination diet, failure to thrive, food hypersensitivity, growth, growth disorders, infant, milk hypersensitivity

## Abstract

**Aim:**

To investigate growth deceleration and associations with clinical manifestations and dietary elimination in infants with gastrointestinal cow's milk allergy (CMA).

**Methods:**

In this case series, infants at a specialised clinic had their clinical and anthropometric data analysed. Weight (WD) and length deceleration (WD and LD) were determined by z‐scores, with reductions > 0.67 classified as failure to thrive (FTT).

**Results:**

Sixty infants (1–7 months) were assessed. WD and LD were identified in 53.3% and 51.1%, respectively. FTT was observed in 26.6% (weight) and 14.9% (length). Only 15.0% were exclusively breastfed, and 41.6% received specialised formula. 73.1% of mothers reported multiple food eliminations. Hematochezia (60.0%) was the most frequent clinical manifestation. WD was associated with full‐term birth (*p* = 0.021; effect size [ES] = 0.3) and higher birth weight z‐score (*p* = 0.001; ES = 0.5). LD was associated with a higher birth length z‐score (*p* = 0.039; ES = 0.3) and marginally associated with diarrhoea (*p* = 0.059; ES = 0.3).

**Conclusion:**

Growth deceleration was highly frequent among infants with CMA, even prior to confirmation. Clinically meaningful trajectory changes occur in the absence of overt undernutrition. Early nutritional assessment at the time of initial clinical suspicion is essential to prevent progressive nutritional impairment.

## Key Notes

- Self‐initiated elimination diets frequently precede referral to specialised care and may adversely affect the nutritional status of infants with suspected cow's milk allergy.
- More than one quarter of infants presented with failure to thrive at the initial assessment, even in the absence of classical wasting criteria.
- Immediate specialised nutritional support at the first clinical suspicion of cow's milk allergy is fundamental to prevent progressive growth deceleration and failure to thrive.

**Abbreviations**
CBF: Complemented Breastfeeding
CMA: Cow's Milk Allergy
EBF: Exclusive Breastfeeding
EH: Extensively Hydrolysed (formula)
ES: Effect Size
FAA: Free Amino Acids (formula)
FTT: Failure to Thrive
L/A: Length‐for‐age
LD: Length Deceleration
OFC: Oral Food Challenge
W/A: Weight‐for‐age
WD: Weight Deceleration
WHO: World Health Organization

## 1 Introduction

The clinical impact of cow's milk allergy (CMA) on nutritional health and child growth is well recognised. In particular, the non‐IgE‐mediated form poses a diagnostic challenge due to its insidious onset, nonspecific gastrointestinal symptoms, and the absence of a specific laboratory marker. In such cases, cow's milk elimination is commonly used as both a diagnostic and therapeutic strategy, with the oral food challenge (OFC) considered the gold standard for confirmation [1, 2, 3].

Several mechanisms can compromise the growth of infants with gastrointestinal manifestations of CMA such as persistent subclinical inflammation, atopic dermatitis, increased intestinal permeability, nutritional deficiencies resulting from feeding difficulties (e.g., inappetence), the elimination of multiple foods, and limitations associated with a milk exclusion diet, especially in cases for which breastfeeding is not possible, as cow's milk becomes the main source of nutrition for the infant [2, 4, 5].

The elimination of cow's milk from the diet is essential to the diagnosis and treatment [6]. However, in everyday clinical practice—particularly in public health settings with limited resources—performing an oral food challenge (OFC) is often unfeasible. As a result, many infants remain on elimination diets for extended periods based solely on clinical criteria. On the other hand, delaying diagnosis favours the adoption by families of inappropriate elimination diets without specialised nutritional supervision, which can exert a negative impact on infant growth [2, 3].

Although this context is worrisome, few studies have investigated the growth of infants with gastrointestinal manifestations of CMA [7, 8, 9] before the confirmation of the diagnosis and the start of structured treatment. Moreover, these studies prioritise the use of z‐score cutoff points to diagnose malnutrition without considering subtler changes, such as progressive declines in z‐scores that do not yet exceed the threshold for malnutrition but are clinically significant.

Evidence suggests that reductions greater than 0.67 in weight or height z‐scores may indicate failure to thrive (FTT) even in infants within the normal range, as this variation constitutes the crossing of relevant percentile bands on the charts of the World Health Organization (WHO) [10, 11, 12]. The early detection of this growth deceleration can enable timely interventions and avoid progression to more severe malnutrition.

Therefore, the present study aimed to investigate the growth deceleration process and its association with clinical manifestations and dietary elimination in infants with gastrointestinal manifestations of cow's milk allergy (CMA).

## 2 Methods

### 2.1 Study Design, Location, and Population

A case series study was conducted involving all infants with gastrointestinal manifestations of cow's milk allergy (CMA) followed up at the paediatric gastroenterology outpatient clinic of a university hospital in the northeast of Brazil, in the period from December 2016 to June 2019. This hospital is a referral centre for the diagnosis and management of CMA, serving mainly patients from families who face significant challenges in accessing specialised care and high‐cost formulas.

The case series was composed of medical records of infants who presented to the outpatient clinic up to seven months of age and with gastrointestinal manifestations of CMA. Infants with neonatal hypoxia or cardiac and renal congenital diseases were excluded.

### 2.2 Clinical Assessment and Diagnostic of CMA

A team of experienced paediatric gastroenterologists assessed and monitored the infants following specific guidelines for the diagnosis and management of CMA [2]. As a referral centre for the Brazilian Public Healthcare System, the service ensures free access to special formulas. This process requires a standardised diagnostic validation through a medical report issued by the certified specialist, with periodic reassessments and ongoing clinical follow‐up.

According to the institutional protocol and in line with the most recent guidelines from the European Society for Paediatric Gastroenterology, Hepatology and Nutrition (ESPGHAN), 2024 [2], the diagnosis was primarily based on clinical history, response to an elimination diet, and symptom recurrence upon reintroduction. Given the retrospective design and the clinical instability of some infants at referral, oral food challenges were not systematically performed or consistently documented, as specific IgE testing or Skin Prick Tests (SPT) were not routinely performed for these phenotypes due to their low diagnostic sensitivity.

A six‐month follow‐up period served as a clinical validation window to ensure diagnostic consistency. Cases in which CMA was ruled out during this follow‐up period were excluded from the analysis.

At the initial assessment in the specialised clinic, therapeutic management was standardised according to the current guideline [2]. Breastfeeding mothers were advised to follow a cow's milk elimination diet with appropriate nutritional counselling to ensure adequacy. Infants requiring breast milk substitutes received specialised formulas—either extensively hydrolysed or amino acid‐based—through the Brazilian public healthcare program. For infants referred previously to unsupervised restrictive diets, the nutritional plan was reassessed and adjusted by a paediatric gastroenterologist.

The clinical manifestations investigated in this study were hematochezia, diarrhoea, vomiting, and atopic dermatitis. The clinical definitions followed standard criteria and were recorded in the medical records by the paediatric gastroenterologist. Hematochezia was defined as the presence of bright red blood in the stool. Vomiting was differentiated from regurgitation by the occurrence of abdominal effort. Diarrhoea was characterised by a change in stool consistency (from pasty to liquid or semi‐liquid), with a frequency of more than three bowel movements per day. Atopic dermatitis was characterised by areas of dry, erythematous, pruritic skin, especially on the cheeks and limbs.

### 2.3 Anthropometric Assessment

Birth anthropometric data were obtained from medical records as reported by parents or caregivers at the first visit. Weight and length at admission were measured by the specialised outpatient team and subsequently retrieved from the medical records for analysis. All results refer to this initial assessment. For each infant, weight and length at birth and at the first specialised consultation were considered, and the interval between these time points corresponded to the infant's age at referral.

The anthropometric measurements performed by paediatric gastroenterologists (specialised team) trained according to the Brazilian guidelines for the anthropometric assessment of infants. Weight was determined on a Filizola Baby digital scale (capacity: 125 g to 15 kg; five‐gram units), and length was determined using a portable infant meter (accuracy: 1 mm; capacity: 105 cm), both of which were available at the outpatient clinic.

The anthropometric diagnosis was based on the length‐for‐age (L/A) and weight‐for‐age (W/A) using the WHO references [13] and AnthroPlus program, version 3.2.2, considering sex, chronological age, and age corrected for prematurity, when necessary. The results were expressed as z‐scores. Growth deceleration was defined by a reduction in the z‐score of the W/A and L/A indices occurring between birth and the initial assessment at the outpatient clinic, leading to the classification of weight deceleration (WD) and length deceleration (LD), respectively. When this negative change exceeded a z‐score of 0.67, failure to thrive (FTT) or catch‐down growth, as defined by Ong et al. [10], was recorded. Cases of wasting and stunting, defined as a z‐score < −2.0 for the respective anthropometric indices, were identified at the initial appointment.

### 2.4 Data Collection, Control, Minimisation of Bias

Data were collected from the clinical records of infants treated at the outpatient clinic using a structured, standardised protocol to ensure the consistency and comparability of the information. Birth‐related, dietary, and anthropometric data recorded in the medical records at the initial assessment were used. Anthropometric measurements (weight and length) were performed with due accuracy by the paediatric gastroenterologists of the outpatient clinic (trained, experienced professionals) following the Brazilian guidelines to minimise the risk of measurement bias. To reduce the occurrence of classification/judgement bias, a clear definition was established to identify cases of infants with CMA. To reduce the risk of selection bias, restrictive adjustment was performed with the adoption of exclusion criteria, as detailed above. The outcomes of the study (WD, LD, and FTT) were assessed after data extraction from the medical records and categorisation, thus minimising the risk of measurement and interpretation bias during the data collection process.

### 2.5 Statistical Analysis

Statistical analyses were performed with the aid of the SPSS 22 software (IBM Corporation, Armonk, NY, USA). The Shapiro–Wilk test was used to determine the normality of the variables. Normally distributed variables were expressed as mean and standard deviation. Non‐normally distributed variables were expressed as median and interquartile range. The Mann–Whitney *U* test was used to compare two independent medians. The effect size was determined using the rank‐biserial correlation. Either Pearson's chi‐square test or, when appropriate, Fisher's exact test was used to determine associations between independent variables and WD/LD. The effect size was estimated using Cramer's V test. The significance level was set at 5% (*p* < 0.05).

### 2.6 Ethics Statement

This study received approval from the Human Research Ethics Committee of *Hospital das Clínicas da Universidade Federal de Pernambuco* (certificate number: 89914425.1.0000.8807).

## 3 Results

**FIGURE 1:**
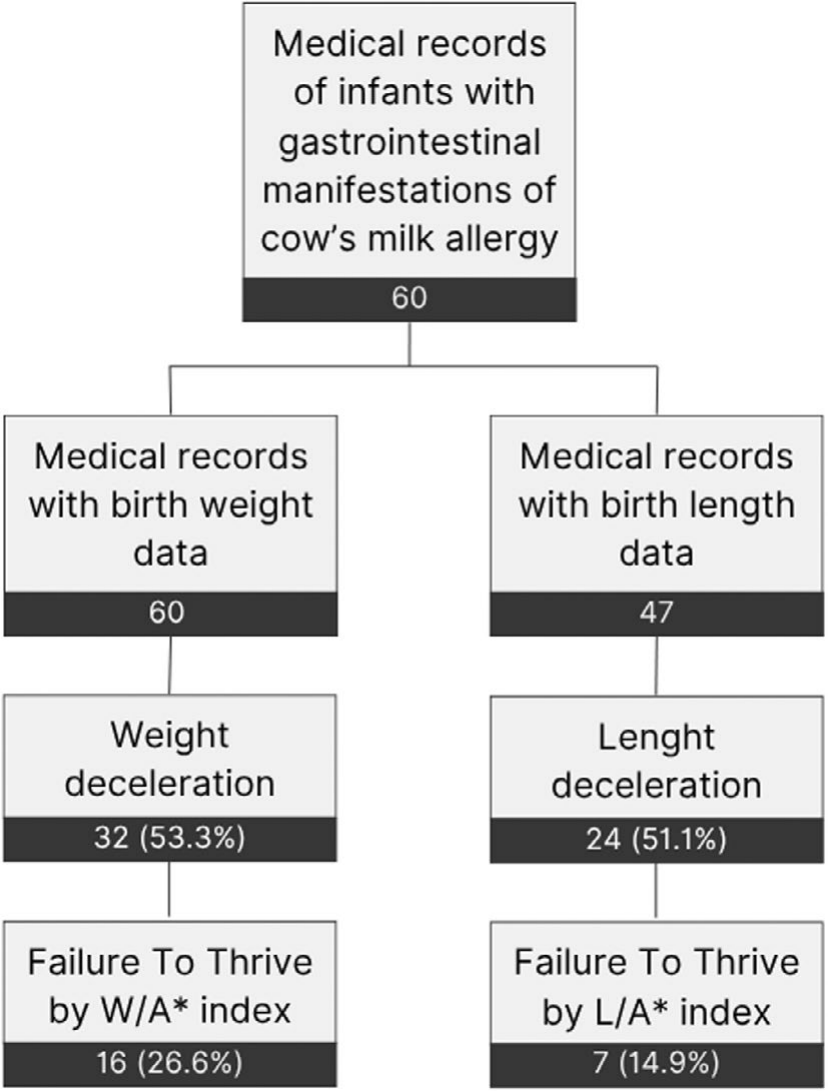
Frequency of growth disorders in infants with gastrointestinal manifestations of cow's milk allergy at initial assessment. W/A = weight‐for‐age; L/A = length‐for‐age.

Sixty infants with gastrointestinal manifestations of CMA up to seven months of age were analysed in the present study. Failure to Thrive (FTT) based on weight was observed in one‐quarter (26.6%), although more than half of the sample already exhibited weight deceleration (WD) at the initial assessment. Similarly, FTT based on length was identified in 14.9% of the infants. But, wasting and stunting were identified in five infants (8.3%) and nine infants (15.0%), respectively. Figure 1 displays the distribution of WD, length deceleration (LD), and FTT based on both parameters.

Among the 34 infants (56.6%) who were breastfeeding at the initial assessment, nine (15%) were exclusively breastfed, and 25 (41.6%) received a complementary specialised formula. Among the breastfeeding mothers, 73.1% excluded milk and at least one other food from their diet. Hematochezia was the most frequent clinical manifestation (36 infants; 60.0%). Other characteristics of the group are displayed in Table 1.

**TABLE 1:** Characteristics of infants (*n* = 60).

Sex (male), *n* (%)	31 (51.7)
Prematurity, *n* (%)	10 (16.7)
Birth weight z‐score†	−0.30 (−1.19–0.52)
Low birth weight^∆^, *n* (%)	6 (10%)
Adequate birth weight, *n* (%)	54 (90%)
Weight‐for‐age z‐score at initial assessment*	−0.38 (± 1.07)
Wasting^∆^, *n* (%)	5 (8.3%)
Adequate weight, *n* (%)	55 (91.7%)
Length‐for‐age z‐score at birth, *n* = 47†	−0.63 (−1.32–0.3)
Short birth length^∆^, *n* (%)	4 (8.5%)
Adequate birth length, *n* (%)	43 (91.5%)
Length‐for‐age z‐score at initial assessment, *n* = 59†	−0.22 (−1.32–0.36)
Stunting^∆^, *n* (%)	9 (15.3%)
Adequate length, *n* (%)	50 (84.7%)
Age at initial assessment (months)*	4.0 (3.0–5.2)
Breastfeeding, *n* (%)	34 (56.6)
Type of formula, *n* = 50	
Amino acid‐based formula, *n* (%)	33 (66.6)
Extensively hydrolyzed formula, *n* (%)	17 (33.4)
Complementary feeding, *n* = 58, *n* (%)	11 (18.9)
Mother's restriction of other foods besides milk, *n* = 26, *n* (%)	19 (73.1)
Quantity of restricted foods, *n* = 26	
Milk and dairy, *n* (%)	7 (26.9)
Milk and dairy +1, *n* (%)	10 (38.5)
Milk and dairy +2, *n* (%)	3 (11.5)
Milk and dairy +3, *n* (%)	5 (19.2)
Milk and dairy +4, *n* (%)	1 (3.9)
Age at start of elimination diet, *n* = 44 (months)*	2.0 (1.0 to 3.0)
Duration of elimination diet, *n* = 44 (months)*	2.0 (1.0 to 3.2)
Hematochezia, *n* (%)	36 (60.0)
Diarrhoea, *n* (%)	29 (48.3)
Vomiting, *n* (%)	16 (26.6)
Atopic dermatitis, *n* (%)	12 (20.0)

^†^ Median (P25 – P75).

* Mean ± standard deviation.

^∆^ Low birth parameters, wasting, and stunting were defined as z‐score < −2 according to WHO standards.

In the bivariate analysis (Table 2), WD was significantly associated with full‐term birth (*p* = 0.021; effect size [ES] = 0.3) and higher birth weight z‐score (*p* = 0.001; ES = 0.5). The variables “restriction of other foods” and “quantity of other foods restricted” had medium effect sizes (ES = 0.3 for both variables), although without statistical significance (*p* = 0.105 and *p* = 0.109, respectively). LD was significantly associated with a higher length at birth z‐score, with a medium effect size (*p* = 0.039; ES = 0.3). Moreover, a tendency toward LD was found in infants with diarrhoea, with a medium ES (*p* = 0.059; ES = 0.3).

**TABLE 2:** Factors associated with weight and length deceleration.

		Weight Deceleration		
		(+)	(−)	
	*n* = 32	*n* = 28	ES^■^	*P*
Sex (male)	19 (61.3%)	12 (38.7%)	0.2*	0.201*
Full‐term birth	22 (53.7%)	19 (46.3%)	0.3^□^	0.021^□^
Birth weight (Kg)†	3.4 (3.0–3.6)	2.9 (2.6–3.1)	0.5^◊^	0.001^◊^
Age at initial assessment (months)†	4.0 (3.0–5.0)	4.0 (3.0–6.0)	0.1^◊^	0.611^◊^
Breastfeeding, *n* (%)	19 (55.8%)	15 (44.2%)	0.1*	0.651*
Type of formula, *n* = 50 (AF^∆^)	18 (54.5%)	15 (45.5%)	0.1*	0.616*
Complementary feeding, *n* = 58	6 (54.5%)	5 (45.5%)	0.0*	0.835*
Restriction of other foods, *n* = 38	12 (63.2%)	7 (36.8%)	0.3*	0.105*
Quantity of other restricted foods, *n* = 38†	1.0 (0.0–2.5)	0.0 (0.0–1.0)	0.3^◊^	0.109^◊^
Age at start of elimination diet, *n* = 44 (months)†	1.5 (1.0–3.0)	2.0 (0.2–3.0)	0.0^◊^	0.990^◊^
Duration of elimination diet, *n* = 44 (months)†	2.0 (1.0–3.0)	2.5 (1.0–3.7)	0.1^◊^	0.679^◊^
Hematochezia	20 (55.6%)	16 (44.4%)	0.1*	0.673*
Diarrhoea	18 (62.1%)	11 (37.9%)	0.2*	0.190*
Vomiting	7 (46.7%)	8 (53.3%)	0.1*	0.550*
Atopic dermatitis	7 (58.3%)	5 (41.7%)	0.1*	0.698*

		Length Deceleration		
		(+)	(−)	
	*n* = 24	*n* = 23	ES	*P*
Sex (male)	14 (58.4%)	10 (41.7%)	0.1*	0.308*
Full‐term birth	19 (55.9%)	15 (44.1%)	0.1^□^	0.727^□^
Birth weight (Kg)†	3.1 (2.8–3.4)	3.2 (2.9–3.6)	0.1^◊^	0.233^◊^
Birth length (cm)†	48.2 (47.0–50.1)	48.0 (46.2–49.2)	0.2^◊^	0.294^◊^
Age at initial assessment (months)†	4.0 (3.0–5.2)	4.0 (3.0–6.0)	0.1^◊^	0.486^◊^
Breastfeeding, *n* (%)	15 (51.7%)	14 (48.3%)	0.0*	0.908*
Type of formula, *n* = 38 (AF^∆^)	14 (51.9%)	13 (48.1%)	0.1*	0.721*
Complementary feeding, *n* = 45	3 (30.0%)	7 (70.0%)	0.2^□^	0.175^□^
Restriction of other foods, *n* = 33	10 (52.6%)	9 (47.4%)	0.1*	0.579*
Quantity of other restricted foods, *n* = 33†	1.0 (0.0–2.2)	1.0 (0.0–1.0)	0.1^◊^	0.557^◊^
Age at start of elimination diet, *n* = 35 (months)†	1.5 (0.2–2.0)	2.0 (0.0–3.0)	0.1^◊^	0.613^◊^
Duration of elimination diet, *n* = 35 (months)†	2.5 (2.0–3.0)	2.0 (1.0–4.0)	0.0^◊^	0.807^◊^
Hematochezia	17 (60.7%)	11 (39.3%)	0.2*	0.108*
Diarrhoea	16 (64.0%)	9 (36.0%)	0.3*	0.059*
Vomiting	6 (66.7%)	3 (33.3%)	0.2*	0.298*
Atopic dermatitis	4 (44.4%)	5 (55.6%)	0.1^□^	0.659^□^

^†^ Median (P25 – P75); ^∆^AF = Amino acid‐based formula.

^■^ ES = effect size.

* Chi‐square test and Cramer's V ES.

^□^ Fisher's exact test and Cramer's V ES.

^◊^ Mann–Whitney test and rank‐biserial ES.

The characteristics of the 16 infants with FTT based on weight are described in Table 3. These infants arrived at the initial assessment on a diet that excluded cow's milk, with a median exclusion time of two months (1.0–3.75). Eleven (68.7%) were breastfeeding, and two (12.5%) were exclusively breastfed.

**TABLE 3:** Characteristics of 16 infants with failure to thrive based on weight‐for‐age index at initial assessment.

Patient	Sex	∆* in weight/age z‐score	∆* in length/age z‐score	Birth weight	Age (months)	Premature birth	Arrived with milk elimination diet	Excludes other types of foods	Age at start of elimination diet	Breastfeeding	Type of formula	Complementary feeding	Feeding	Associated gastrointestinal clinical manifestations	Atopic dermatitis	Hed infections before first assessment
1	F^‡^	−2.07	2.24	4.11	4	No	Yes	No	3	Yes	EH^†^	No	CBF^¤^	Hematochezia and diarrhoea	No	No
2	M^£^	−2.01	NI	3.50	3	No	Yes	No	NI	No	EH	No	Formula	Diarrhoea	Yes	No
3	F	−1.92	NI	3.83	5	No	Yes	No	1	Yes	EH	Yes	CBF	Hematochezia	No	No
4	M	−1.39	0.88	3.20	2	No	Yes	NI^∆^	0	Yes	FAA^¥^	No	CBF	None	Yes	No
5	M	−1.34	−1.07	2.83	3	No	Yes	3	1	Yes	None	No	EBF^§^	Vomiting	No	No
6	M	−1.26	−1.35	3.78	3	No	Yes	1	1	Yes	FAA	No	CBF	Hematochezia, diarrhoea and vomiting	No	Yes
7	M	−1.16	NI	2.68	4	Yes	NI	NI	4	No	EH	No	Formula	Hematochezia and Vomiting	No	No
8	F	−1.03	NI	3.55	5	No	Yes	No	2	No	FAA	No	Formula	Hematochezia and Vomiting	Yes	No
9	M	−0.91	−1.71	3.60	2	No	Yes	1	1	Yes	EH	No	CBF	Hematochezia and diarrhoea	No	No
10	M	−0.89	1.16	2.85	5	No	Yes	NI	NI	No	FAA	No	Formula	Vomiting	No	No
11	M	−0.85	−0.54	3.94	5	No	Yes		4	No	FAA	No	Formula	Hematochezia, diarrhoea and vomiting	No	Yes
12	F	−0.8	0.57	3.90	4	No	Yes	1	0	Yes	FAA	No	CBF	None	Yes	No
13	M	−0.74	−0.06	3.65	6	No	Yes	No	5	Yes	FAA	NI	CBF	Hematochezia and diarrhoea	No	No
14	M	−0.72	2.16	3.63	4	No	Yes	1	2	Yes	None	No	EBF	Hematochezia	No	No
15	M	−0.71	−0.37	3.17	6	No	Yes	NI	0	Yes	FAA	Yes	CBF	Hematochezia	No	No
16	F	−0.68	2.14	3.50	6	No	Yes	4	0	Yes	FAA	Yes	CBF	Hematochezia and diarrhoea	No	No

Abbreviations: F^‡^ = female; M^£^ = male. NI^∆^ = no information. CBF^¤^ = complemented breastfeeding. EBF^§^ = exclusive breastfeeding.

* ∆ = z‐score at initial assessment – z‐score at birth.

^¥^ FAA = free amino acids.

^†^ EH = extensively hydrolysed.

## 4 Discussion

This study revealed a high frequency of weight deceleration and length deceleration among infants up to seven months of age with gastrointestinal manifestations of CMA. By using the World Health Organization (WHO) growth standards as a normative international reference, we compared our sample's trajectories against the expected growth of healthy, breastfed infants. This approach served as a robust external control, allowing us to identify deviations from optimal growth patterns. These findings suggest a relevant association between clinically suspected predominantly non‐IgE‐mediated CMA and early growth deceleration, although causality cannot be inferred due to the observational design.

Failure to thrive (FTT) based on weight was found in more than one‐quarter of the participants, demonstrating nutritional impairment before the possibility of the diagnosis, although only 8.3% were classified as underweight (z‐score < −2.0). This underscores that fixed cutoff points underestimate nutritional risk. The z‐score variation > 0.67 adopted in this study is supported by studies stating it enables recognition of clinically relevant growth problems, as it corresponds to crossing a percentile band on the growth curve, representing a clinically significant deviation from the expected healthy trajectory [11, 12]. Additionally, the higher proportion of infants already exhibited a clear downward trend in their growth trajectories—WD in more than half—highlights the importance of assessing growth velocity to favour timely interventions before wasting or stunting.

The association between WD and a higher birth weight is compatible with evidence that well‐nourished infants at birth tend to have slower growth rates than smaller infants when experiencing inflammatory processes or an inadequate diet [14, 15]. This also explains the significant association with a full‐term birth, as preterm infants tend to have lower birth weights. These findings underscore the importance of considering birth characteristics as part of the risk assessment for growth deceleration, even in healthy infants.

Among dietary factors, the elimination of multiple foods by mothers had a moderate effect on WD. As most infants were either exclusively or partially breastfed, such eliminations may have compromised the nutritional composition of the breast milk. The literature states that the mother's diet directly influences the nutritional quality of breast milk, potentially affecting the supply of macro‐ and micronutrients essential to the nutrition of allergic infants [16, 17, 18]. As the *p*‐value is affected by sample size [19, 20], the association between the elimination of multiple foods in lactating women and WD may achieve statistical significance in studies with greater sample power.

Many infants arrived at the clinic already using special formulas, often amino acid‐based, without a clear clinical indication. This pattern indicates the adoption of restrictive dietary practices prior to a specialised assessment, possibly motivated by visible clinical manifestations and difficulty in gaining prompt access to specialised care. In low‐income contexts, such as that of the population studied, limited access to these formulas can aggravate nutritional vulnerability and compromise the adequate management of the condition (Assis et al. [9]).

Symptoms such as hematochezia, although clinically mild, can trigger apprehension in caregivers and lead to hasty, unsupervised feeding decisions [21]. In the present study, hematochezia (60.0%) showed an effect on growth deceleration similar to that of vomiting. This is supported by Rosow et al., who reported that infants with allergic proctocolitis exhibited significant alterations in their growth trajectory, suggesting that even ‘mild’ phenotypes may serve as indirect markers of nutritional risk when associated with inappropriate dietary behaviours [22].

Our findings suggest that the growth deceleration observed at the initial assessment was largely related to the period preceding specialised care. In primary care settings without structured support, parental insecurity may lead to self‐directed and potentially harmful dietary practices, including inappropriate discontinuation of breastfeeding or unsupervised use of specialised formulas. In socially vulnerable populations, the high cost of hypoallergenic formulas combined with limited access to specialised follow‐up represents a substantial barrier to adequate management.

The effectiveness of our centre's structured approach in improving the nutritional status of infants with CMA through specialised follow‐up has been previously demonstrated (Assis et al. [9]). Taken together, these findings indicate that limited nutritional guidance and restricted access to appropriate formulas likely contributed to suboptimal energy intake and subsequent growth deceleration at admission. This supports the hypothesis that early nutritional compromise may be a key component of the “diagnostic and therapeutic gap” occurring prior to specialised intervention.

A trend toward an association was observed between diarrhoea and length deceleration (*p* = 0.059), with a moderate effect size. This finding is consistent with previous reports, as diarrhoea is typically associated with more severe and inflammatory gastrointestinal phenotypes of cow's milk allergy, which may exert a greater impact on intestinal absorption and linear growth [2, 3].

The specific analysis of the 16 infants with FTT based on weight revealed that most were already on an elimination diet, which had been in place for approximately two months prior to arrival at the clinic, possibly without nutritional monitoring and contributing to impaired growth. Only 12.5% were exclusively breastfed, which may indicate the early introduction of formulas and reinforce their inappropriate use. Although quantitative energy intake was not directly assessed due to the retrospective design, this context suggests that inappropriate formula dilution—often adopted as a cost‐saving strategy in socioeconomically vulnerable families—or delayed introduction of complementary feeding due to parental insecurity may have led to a sustained caloric deficit, thereby contributing to the observed growth impairment. More than one‐third also had low length, suggesting that the period of exclusion without guidance may have impacted not only weight but also linear growth.

The investigation of data from the initial assessment—when CMA is only a suspected diagnosis—demonstrates the importance of early intervention strategies motivated by the difficulties faced in confirming the diagnosis through oral food challenge testing, which, although considered the gold standard, is not always feasible in clinical practice. Moreover, a delayed diagnosis can perpetuate inappropriate behaviours, thus exerting a negative impact on growth [2, 3]. This underscores the importance of an early preventive approach, with an emphasis on individualised nutritional care beginning with the first clinical signs.

Among the study's limitations, the absence of diagnostic confirmation via oral food challenge—considered the diagnostic gold standard—stands out. However, the six‐month follow‐up period served as a clinical validation window, enhancing diagnostic consistency. The retrospective design precluded precise quantitative assessment of daily energy intake, including the exact volumes and caloric density of foods consumed prior to the initial evaluation. Future prospective studies incorporating structured dietary assessments are warranted to better elucidate the relationship between energy intake and growth outcomes.

The delay between the onset of clinical symptoms and a specialised evaluation—which we could characterise as a ‘diagnostic gap’—appears to be a critical period for nutritional compromise. Since most infants in our series reached the outpatient clinic already on an elimination diet for a median of two months, and the growth deceleration was often already established. This scenario highlights that, in clinical practice, the focus should not only be on the formal diagnostic confirmation through an oral food challenge, but also on the immediate nutritional protection of the infant as soon as CMA is suspected. Waiting for diagnostic stability to perform a gold‐standard test in an infant already showing a downward growth trajectory may miss the window for preventing failure to thrive.

Despite the limitations of this study, such as the small size of the case series, the findings make important practical and scientific contributions. In clinical practice, the data reveal the need for nutritional monitoring beginning with the first signs of CMA, even before diagnostic confirmation, with special attention to indicators at birth and clinical manifestations, even if mild. Criteria sensitive to FTT should be used to detect nutritional risks early in infants with gastrointestinal symptoms. From a public policy perspective, the findings highlight the importance of ensuring timely access to specialised formulas and adequate nutritional monitoring, especially for vulnerable populations. Educational strategies for healthcare providers and caregivers are also crucial to the prevention of inappropriate dietary exclusions.

In summary, the data from the present study indicate that failure to thrive—in the form of clinically significant growth deceleration—is highly prevalent among infants with gastrointestinal forms of cow's milk allergy, especially in the context of socioeconomic vulnerability. FTT can occur even in the absence of wasting or stunting, defined by a z‐score cutoff point of < −2.0. Moreover, restrictive dietary practices adopted early without specialised guidance have a greater effect on WD than clinical manifestations. Our findings highlight the need for clinical protocols that include longitudinal growth monitoring as a sensitive tool for early detection of nutritional risk, especially in infants undergoing diagnostic evaluation for CMA. Future analytical studies with formal diagnostic confirmation are needed to further explore these associations and support evidence‐based guidelines.

## 5 Conclusion

Growth deceleration was frequently observed among infants with gastrointestinal manifestations of CMA followed in a specialised outpatient clinic. The assessment of growth curves and z‐score variation over time allowed for the sensitive detection of subtle but progressive changes associated with the clinical presentation. This pattern of early anthropometric compromise highlights the importance of nutritional monitoring even in the absence of diagnostic confirmation, supporting clinical management in contexts of diagnostic uncertainty.

## Author Contributions

Priscila Prazeres de Assis: Principal investigator; responsible for the study design and planning, data analysis, interpretation and discussion, as well as drafting and critical revision of the final manuscript. Margarida Maria de Castro Antunes: Co‐supervisor of the research; contributed to the article planning, study supervision, data interpretation, and critical revision of the final manuscript. Alcides da Silva Diniz: Contributed to the study guidance, data interpretation, and critical revision of the final manuscript. Adrielle Cavalcanti de Pontes Araújo: Contributed to the study guidance and critical revision of the final manuscript. Camila de Souza Rêgo: Contributed to the study guidance and critical revision of the final manuscript. Poliana Coelho Cabral: Main supervisor of the research; involved in the study design and planning, supervision, data analysis and interpretation, and critical revision of the final manuscript. All authors critically revised the manuscript, agreed to be fully accountable for ensuring the integrity and accuracy of the work, and read and approved the final manuscript.

## Funding

This work was supported by the Coordenação de Aperfeiçoamento de Pessoal de Nível Superior (88887.984623/2024‐00). This research is supported by a doctoral scholarship provided by the Coordination for the Improvement of Higher Education Personnel (CAPES), Brazil.

## Conflicts of Interest

The authors declare no conflicts of interest.

## Data Availability

The data that support the findings of this study are available on request from the corresponding author. The data are not publicly available due to privacy or ethical restrictions.
